# Clinical Subgroups in Bilateral Meniere Disease

**DOI:** 10.3389/fneur.2016.00182

**Published:** 2016-10-24

**Authors:** Lidia Frejo, Andres Soto-Varela, Sofía Santos-Perez, Ismael Aran, Angel Batuecas-Caletrio, Vanesa Perez-Guillen, Herminio Perez-Garrigues, Jesus Fraile, Eduardo Martin-Sanz, Maria C. Tapia, Gabriel Trinidad, Ana María García-Arumi, Rocío González-Aguado, Juan M. Espinosa-Sanchez, Pedro Marques, Paz Perez, Jesus Benitez, Jose A. Lopez-Escamez

**Affiliations:** ^1^Otology and Neurotology Group CTS495, Department of Genomic Medicine – Centro de Genómica e Investigación Oncológica – Pfizer/Universidad de Granada/Junta de Andalucía (GENYO), Granada, Spain; ^2^Department of Otorhinolaryngology, Division of Otoneurology, Complexo Hospitalario Universitario, Santiago de Compostela, Spain; ^3^Department of Otolaryngology, Complexo Hospitalario de Pontevedra, Pontevedra, Spain; ^4^Department of Otolaryngology, Hospital Universitario Salamanca, Salamanca, Spain; ^5^Department of Otorhinolaryngology, Hospital Universitario La Fe, Valencia, Spain; ^6^Department of Otolaryngology, Hospital Miguel Servet, Zaragoza, Spain; ^7^Department of Otolaryngology, Hospital Universitario de Getafe, Getafe, Spain; ^8^Department of Otorhinolaryngology, Instituto Antolí Candela, Madrid, Spain; ^9^Department of Otorhinolaryngology, Division of Otoneurology, Complejo Hospitalario Badajoz, Badajoz, Spain; ^10^Department of Otorhinolaryngology, Hospital Universitario Vall d’Hebron, Barcelona, Spain; ^11^Department of Otorhinolaryngology, Hospital Universitario Marqués de Valdecilla, Santander, Cantabria, Spain; ^12^Department of Otorhinolaryngology, Hospital San Agustin, Linares, Jaen, Spain; ^13^Department of Otorhinolaryngology, Centro Hospitalar de São João, EPE, University of Porto Medical School, Porto, Portugal; ^14^Department of Otorhinolaryngology, Hospital Cabueñes, Gijón, Spain; ^15^Department of Otolaryngology, Hospital Universitario de Gran Canaria Dr. Negrin, Las Palmas, Spain; ^16^Department of Otolaryngology, Instituto de Investigación Biosanitaria ibs.GRANADA, Complejo Hospitalario Universidad de Granada (CHUGRA), Granada, Spain

**Keywords:** cluster analysis, vestibular disorders, hearing loss, tinnitus, Meniere’s disease, migraine, autoimmune disorders, inner ear

## Abstract

Meniere disease (MD) is a heterogeneous clinical condition characterized by sensorineural hearing loss, episodic vestibular symptoms, and tinnitus associated with several comorbidities, such as migraine or autoimmune disorders (AD). The frequency of bilateral involvement may range from 5 to 50%, and it depends on the duration of the disease. We have performed a two-step cluster analysis in 398 patients with bilateral MD (BMD) to identify the best predictors to define clinical subgroups with a potential different etiology to improve the phenotyping of BMD and to develop new treatments. We have defined five clinical variants in BMD. Group 1 is the most frequently found, includes 46% of patients, and is defined by metachronic hearing loss without migraine and without AD. Group 2 is found in 17% of patients, and it is defined by synchronic hearing loss without migraine or AD. Group 3, with 13% of patients, is characterized by familial MD, while group 4, that includes 12% of patients, is associated by the presence of migraine in all cases. Group 5 is found in 11% of patients and is defined by AD. This approach can be helpful in selecting patients for genetic and clinical research. However, further studies will be required to improve the phenotyping in these clinical variants for a better understanding of the diverse etiological factors contributing to BMD.

## Introduction

Meniere’s disease (MD) is a long-lasting disorder of the inner ear characterized by episodes of vertigo lasting from 20 min to hours, low-to-middle frequencies sensorineural hearing loss (SNHL, Table 1), tinnitus, and aural fullness (1). MD patients have phenotypic heterogeneity (2), and it is difficult to define the outcome of the disease in its early stages. Although the frequency of the spells of vertigo is typically greater during the earlier years (3–5), balance problems are observed during the course of the disease and might become severe if patients progress to a bilateral vestibular hypofunction (6, 7). Most of the patients start with SNHL in one ear, and it can appear in the other after several years (metachronic SNHL) (8), but a significant number of individuals show simultaneous SNHL (synchronic SNHL). Bilateral involvement is a major concern for patients because of the loss of vestibular function, and bilateral SNHL has a significant influence in the health-related quality of life in MD patients (9).

**Table 1:** **List of abbreviations**.

AAO-HNS	American Academy Otolaryngology – Head Neck Surgery
AD	Autoimmune disorders/disease
AIED	Autoimmune inner ear disorder
BMD	Bilateral Meniere disease
BMD type 1	Metachronic hearing loss
BMD type 2	Synchronic hearing loss
BMD type 3	Familial Meniere disease
BMD type 4	Meniere disease + migraine
BMD type 5	Meniere disease + autoimmune disease
FMD	Familial Meniere disease
MD	Meniere disease
MRI	Magnetic resonance imaging
RCT	Randomized clinical trials
SNHL	Sensorineural hearing loss
SMD	Sporadic Meniere disease

Several studies have reported contralateral ear involvement between 2 and 73% of cases, depending on the interval of follow-up and the diagnostic criteria used. However, if bilateral MD (BMD) was defined as the combination of clinical symptoms and audiometric tests, the frequency would be 2–47% (7). Some studies describe an interval of 5 years where the incidence was 10–35% (8, 10–13), while in other studies, with a follow-up of 10 years or more, the frequency of BMD ranges from 20 (14–16) to 46% (17). However, more than 20 years of follow-up have also been described, and the incidence rate of bilaterality rises up to 47% (18–22). Although there is a great disparity in the percentage of individuals with bilateral involvement, most of the studies highlighted that the number of patients with contralateral ear involvement increased with the duration of the disease (18, 21, 22).

Several comorbidities have been associated with MD, including autoimmune disorders and migraine. So, MD has been previously associated with several autoimmune diseases, such as systemic lupus erythematous, psoriasis, or rheumatoid arthritis (6, 23), and autoimmunity has been suggested as a potential cause in MD (24) relying on the results of proteomic studies achieved in small series of patients (24–26). However, high levels of circulating immune complexes were not found in most of the patients with MD (27). Furthermore, autoimmune mechanisms seem to be associated with the pathogenesis of some types of SNHL (28, 29), such as sudden SNHL (30), promptly progressive bilateral SNHL (31), and MD (32–34). Additionally, several genes of the immune system have been studied in case–control studies (35–38), but they have not been replicated. Moreover, some data suggest that allelic variants of *MICA* and *TLR10* genes, involved in the innate immune response, may influence the susceptibility and time course of hearing loss of MD in European population (39, 40).

Migraine has been consistently found to be more common in MD than in the general population in case–control studies (41), but it is not clear if this association has any role in the pathophysiology of MD. Vestibular migraine (VM), the condition of episodic vestibular symptoms linked to migraine spectrum (42), may occur in some patients concomitantly with MD (43).

Genetic factors are probably relevant in a subset of patients with MD. So, familial MD was first described in 1949 by Brown (44), and many studies have described familial cases of MD (45). The genetic contribution to MD has been recently reviewed (46, 47), and there are several evidences to support a genetic origin in MD. These evidences include (a) the prevalence is higher in European descent population than in Asian (48) or African populations (49) and (b) familiar aggregation has been observed in 6% in South Korea and 8–9% in Spain (2), being *DTNA* and *FAM136A* genes involved in autosomal dominant familial MD (50).

The aim of this study is to describe the phenotype of patients with BMD, including comorbidities such as autoimmune diseases or familial aggregation, and to perform a cluster analysis to identify clinical variants in BMD.

## Materials and Methods

### Subjects

A multicenter, cross-sectional retrospective study was designed, including patients with BMD diagnosed and tracked by the Meniere’s Disease Consortium. For this, the clinical records of a total of 405 patients diagnosed with definite BMD from 16 clinical centers in Spain and Portugal were reviewed in March 2016. MD diagnosis was established according to the diagnostic scale of the American Academy of Otolaryngology Head and Neck Surgery (AAO-HNS) (51). Familial MD (FMD) was defined if at least another relative (first or second degree) fulfilled all the criteria of definite or probable MD, according to the criteria established by the Barany Society International Classification for Vestibular Disorders (1). Patients with unilateral MD or bilateral BMD with less than 5 years of evolution were excluded of the study. Seven patients were excluded because of inconsistent data. This study was approved by the Institutional Review Board for Clinical Research (PI-13-1242).

Every patient underwent a complete neuro-otological evaluation, including a pure-tone audiometry, an otoscopy, nystagmus examination, and a caloric testing. A brain MRI was performed to exclude any other possible cause of neurological symptoms. Patients with simultaneous SNHL in both ears were considered to have synchronic SNHL, while metachronic SNHL was considered if an interval longer than 1 month between the first and the second ear was observed.

Clinical variables studied were as follows: gender, duration of disease, age of onset, family history of MD, hearing loss at diagnosis, hearing stage defined as four-tone average of 0.5, 1, 2, and 3 kHz according to the AAO-HNS criteria (stage 1, ≤25 dB; stage 2, 26–40 dB; stage 3, 41–70 dB, and stage 4, >70 dB), type of headache (migraine, tension-type headache), history of autoimmune disease (AD), cardiovascular risk factors (high blood pressure, type 2 diabetes, dyslipidemia, and smoking), Tumarkin crisis, and the Functional Scale of the AAO-HNS.

### Statistical Analysis

A descriptive statistical analysis was carried out using SPSS software v.22 (SPSS Inc., Chicago, IL, USA). Data are shown as means with their SDs. Quantitative variables were compared using Student’s unpaired *T*-test. Qualitative variables were compared using crosstabs and Fisher’s exact test. Nominal *p*-values <0.05 were considered statistically significant.

We carried out a two-step cluster analysis using log-likelihood distance measures, which can detect relationships within a complex dataset between patients with multiple distinct characteristics. It tries to identify homogenous groups of cases based on the distribution of some variables (input variables). The method identifies the groups by running pre-clustering first and then by using hierarchical methods to classify and to find the optimal number of clusters.

Initially, we selected variables showing differences between the clinical groups during the descriptive analysis to test its relevance as predictors of clusters. The procedure was iterated several times until we found the minimum number of homogenous clusters. The final cluster analysis was applied using the four following categorical variables: history of autoimmune disease, onset of hearing loss (synchronic/metachronic), FMD or sporadic cases, and migraine. The four variables included produced a silhouette of cohesion and division of 0.8, indicative of good data partitioning. Two additional variables were added to the model: age of onset <40 years old and gender, although their contribution to refine the clustering was limited.

## Results

**Figure 1:**
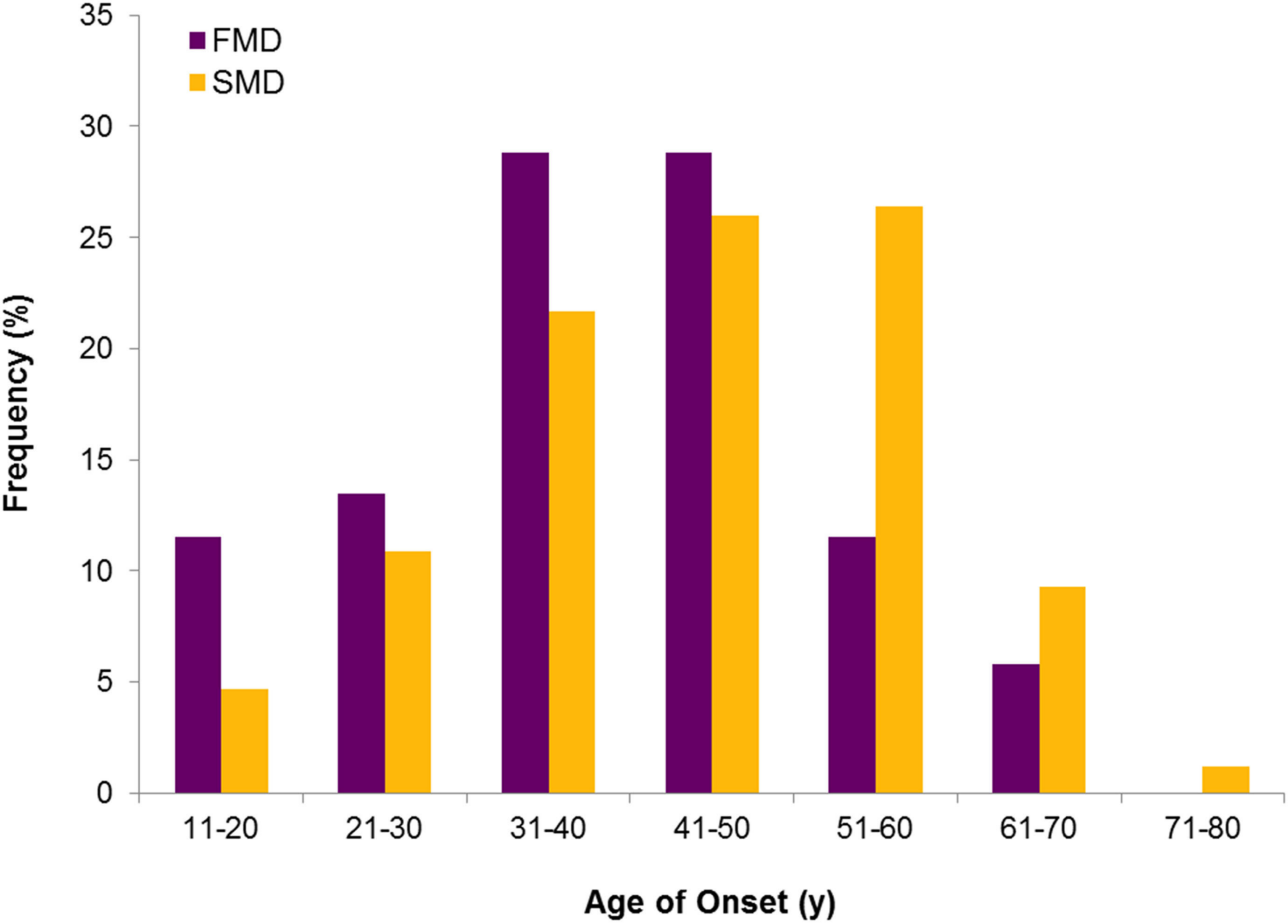
**Age of onset in bilateral Meniere disease**. Distribution of frequencies in familial and sporadic cases shows an earlier onset in FMD.

Three hundred ninety-eight patients with BMD were included in the study. There were 258 sporadic cases and 52 individuals with FMD (20%). Although apparently there were no clinical differences in the phenotype between sporadic and familial cases, FMD had an earlier age of onset (*p* = 0.003) and a higher prevalence of autoimmune comorbidities (Table 2). So, the distribution of frequencies for the age of onset showed that the number of patients starting before 40 years old was significantly higher in the FMD (Figure 1). Table 3 lists the autoimmune comorbid conditions found, being rheumatoid arthritis the most common in our cohort.

**Table 2:** **Clinical phenotype in sporadic and familial Meniere disease with at least 5 years since the onset of the disease**.

Variables	FMD (*n* = 52)	SMD (*n* = 258)	*p*-value
Age, mean (SD)	55.5 (12.7)	61.5 (11.1)	**0.001**
Gender (% women)	34 (65.4)	147 (57.0)	0.28
Age of onset (SD)	39 (12.9)	44.8 (13.1)	**0.003**
Age of onset ≤40, *n* (%)	28 (53.8)	96 (37.2)	**0.03**
Time course (years), mean (SD)	16.3 (8.7)	16.3 (9.4)	0.96
Synchronic, *n* (%)	11 (21.6)	72 (27.9)	0.39
Metachronic, *n* (%)	40 (78.4)	186 (72.1)	
Hearing loss at diagnosis, mean (SD)	51.9 (15.5)	56.6 (17.8)	0.092
Headache, *n* (%)	23 (44.2)	92 (36.1)	0.27
Migraine, *n* (%)	13 (25.0)	44 (17.3)	0.24
Rheumatoid history, *n* (%)	10 (20.4)	25 (9.8)	**0.048**
Hearing stage, *n* (%)			
1	0 (0.0)	4 (1.6)	0.58
2	9 (17.6)	35 (13.6)	
3	28 (54.9)	131 (51.0)	
4	14 (27.5)	87 (33.9)	
Cardiovascular risk			
High blood pressure, *n* (%)	13 (26.5)	93 (39.7)	0.1
Dyslipidemia, *n* (%)	21 (42.0)	111 (47.6)	0.53
Type 2 diabetes, *n* (%)	12 (24.0)	41 (17.4)	0.32
Smoking, *n* (%)	15 (30)	53 (21.5)	0.2
Tumarkin crisis, *n* (%)	17 (35.4)	63 (25.5)	0.16
Functional Scale, *n* (%)			
1	9 (17.6)	53 (21.3)	0.81
2	15 (29.4)	71 (28.5)	
3	10 (19.6)	58 (23.3)	
4	7 (13.7)	35 (14.1)	
5	7 (13.7)	25 (10.0)	
6	3 (5.9)	7 (2.8)	

*SMD, sporadic Meniere disease; FMD, familial Meniere disease*.

*Significant p values in bold*.

**Table 3:** **Autoimmune diseases and other rheumatoid conditions observed in patients with bilateral Meniere disease**.

Autoimmune diseases	*N*
Rheumatoid arthritis	10
Fibromyalgia	6
Arthrosis	5
Ankylosing spondylitis	5
Psoriasis	4
Hypothyroidism	3
Sjogren syndrome	3
Type 1 diabetes	2
Rosacea	2
Graves–Basedow disease	2
Systemic lupus erythematous	2
Psoriatic arthritis	1
Autoimmune inner ear disease	1
Polymyalgia rheumatica	1
Inflammatory bowel disease	1
Cogan syndrome	1
Hip synovitis	1
Carpal tunnel syndrome	1
Undetermined	10

The clinical features in patients with sporadic and FMD were stratified according to the presence or absence of autoimmune comorbidities. In the sporadic cases, headache and migraine were most commonly observed in patients with autoimmune background (62.5 and 33%, respectively) compared with patients without autoimmune comorbidities (33 and 16%), suggesting a potential association between migraine and autoimmunity in patients with sporadic BMD (Table 4).

**Table 4:** **Clinical features of sporadic and familial bilateral Meniere disease stratified by the presence of autoimmune disease (AD)**.

Variables	Sporadic MD			Familial MD		
	AD+ (*n* = 25)	AD− (*n* = 230)	*p*-value	AD+ (*n* = 10)	AD− (*n* = 39)	*p*-value
Age, mean (SD)	61.7 (9.1)	61.6 (11.2)	0.94	56.5 (13.8)	55 (12.4)	0.74
Gender (% women)	18 (72.0)	128 (55.7)	0.14	6 (60.0)	26 (66.7)	0.72
Age of onset (SD)	43.4 (11.0)	45.2 (13.2)	0.5	35.9 (12.3)	40.5 (13.0)	0.31
Age of onset ≤40, *n* (%)	11 (44.0)	82 (35.7)	0.51	6 (60.0)	19 (48.7)	0.73
Time course (years), mean (SD)	17.4 (8.7)	16.1 (9.6)	0.52	20.7 (8.9)	14 (7.0)	**0.01**
Hearing loss at diagnosis, mean (SD)	57.5 (18.3)	56.7 (17.8)	0.83	52.3 (15.2)	52 (15.9)	0.96
Headache, *n* (%)	15 (62.5)	77 (33.5)	**0.007**	8 (80.0)	14 (35.9)	**0.03**
Migraine, *n* (%)	8 (33.3)	36 (15.7)	**0.044**	5 (50.0)	7 (17.9)	**0.05**
Hearing stage, *n* (%)						
1	0 (0.0)	4 (1.7)	0.37	0 (0.0)	0 (0.0)	0.32
2	5 (20.0)	30 (13.1)		1 (11.1)	8 (20.5)	
3	9 (36.0)	119 (52.0)		4 (44.4)	23 (59.0)	
4	11 (44.0)	76 (33.2)		4 (44.4)	8 (20.5)	
Cardiovascular risk factors						
High blood pressure, *n* (%)	13 (59.1)	80 (37.7)	0.07	2 (20.0)	10 (27.0)	1
Dyslipidemia, *n* (%)	12 (50.0)	97 (46.9)	0.83	4 (40.0)	16 (42.1)	1
Type 2 diabetes, *n* (%)	8 (33.3)	33 (15.8)	**0.046**	5 (50.0)	7 (18.4)	0.09
Smoking, *n* (%)	6 (28.6)	47 (21.0)	0.41	3 (30.0)	12 (30.8)	1
Tumarkin crisis, *n* (%)	6 (27.3)	57 (25.3)	0.8	5 (50.0)	12 (32.4)	0.46
Functional Scale, *n* (%)						
1	4 (17.4)	48 (21.3)	0.94	2 (20.0)	7 (17.9)	**0.007**
2	7 (30.4)	64 (28.4)		4 (40.0)	11 (28.2)	
3	6 (26.1)	52 (23.1)		1 (10.0)	8 (20.5)	
4	2 (8.7)	33 (14.7)		0 (0.0)	6 (15.4)	
5	3 (13.0)	22 (9.8)		0 (0.0)	7 (17.9)	
6	1 (4.3)	6 (2.7)		3 (30.0)	0 (0.0)	

*Significant p values in bold*.

**Figure 2:**
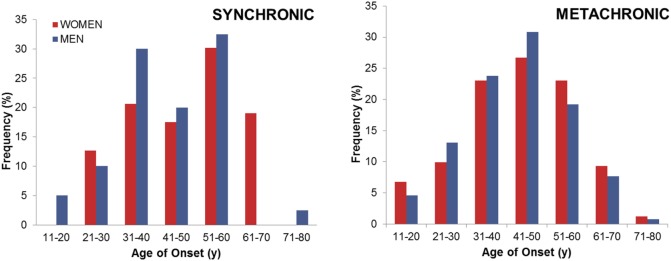
**Age of onset in bilateral Meniere disease according to the type of hearing loss observed**.

We also compared patients according to the onset of hearing loss (Table 5). One hundred three (26%) individuals developed simultaneous hearing loss in both ears (synchronic hearing loss, either symmetric or asymmetric), while 291 (73%) patients started with hearing loss in one ear and developed the hearing loss in the contralateral ear (metachronic hearing loss). Figure 2 compares the distribution of frequencies for the age of onset in patients with synchronic or metachronic hearing loss. There were no clinical differences between them, but the occurrence of headache was most commonly observed in synchronic hearing loss (*p* = 0.0004), and the worst hearing stage was observed in patients with metachronic hearing loss (*p* = 0.004).

**Table 5:** **Clinical features in bilateral Meniere disease according to the onset of hearing loss**.

Variables	Synchronic (*n* = 103)	Metachronic (*n* = 291)	*p*-value
Age, mean (SD)	61 (11.0)	60.1 (11.9)	0.49
Gender (% women)	63 (61.2)	161 (55.3)	0.36
Age of onset (SD)	46.1 (12.8)	43.5 (13.2)	0.07
Age of onset ≤40, *n* (%)	39 (37.9)	118 (40.5)	0.73
Time course (years), mean (SD)	14.4 (8.9)	16.2 (8.9)	0.08
Family history, *n* (%)	39 (39.8)	119 (43.0)	0.64
FMD, *n* (%)	11 (13.3)	40 (17.7)	0.39
Hearing loss at diagnosis, mean (SD)	55.1 (17.0)	55.9 (17.0)	0.71
Headache, *n* (%)	55 (53.4)	96 (33.3)	**0.0004**
Migraine, *n* (%)	25 (24.3)	49 (17.0)	0.11
Rheumatoid history, *n* (%)	15 (15.0)	35 (12.2)	0.49
Hearing stage, *n* (%)			
1	1 (1.0)	6 (2.1)	**0.004**
2	27 (26.5)	34 (11.7)	
3	42 (41.2)	152 (52.4)	
4	32 (31.4)	98 (33.8)	
Cardiovascular risk			
High blood pressure, *n* (%)	47 (51.1)	109 (39.9)	0.068
Dyslipidemia, *n* (%)	53 (55.2)	121 (45.1)	0.097
Type 2 diabetes, *n* (%)	13 (13.5)	50 (18.5)	0.35
Smoking, *n* (%)	22 (21.8)	68 (24.5)	0.68
Tumarkin crisis, *n* (%)	24 (25.8)	69 (24.9)	0.89
Functional Scale, *n* (%)			
1	14 (14.0)	73 (26.0)	0.11
2	29 (29.0)	77 (27.4)	
3	26 (26.0)	55 (19.6)	
4	12 (12.0)	40 (14.2)	
5	16 (16.0)	27 (9.6)	
6	3 (3.0)	9 (3.2)	

*Significant p values in bold*.

**Figure 3:**
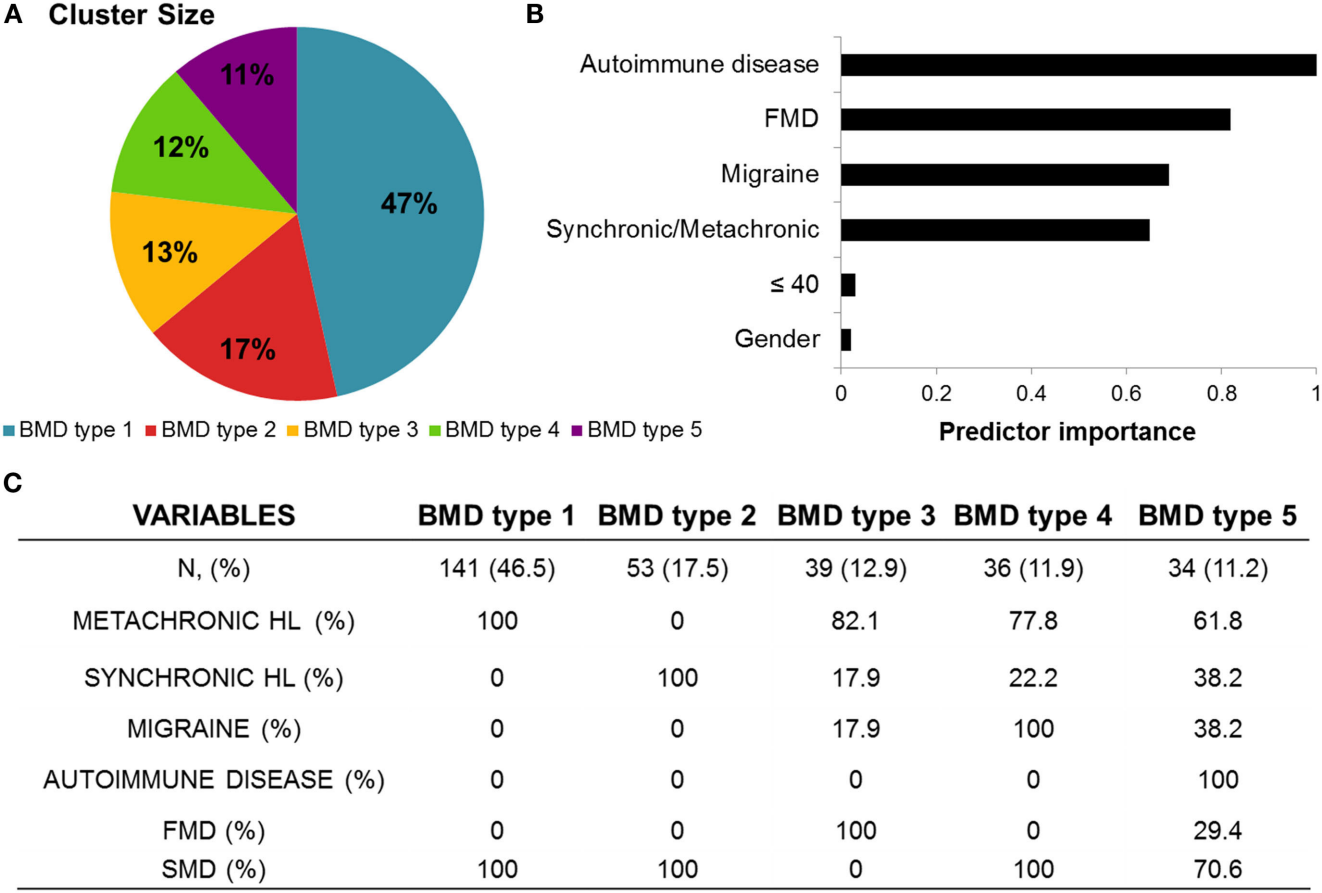
**Summary of cluster analysis in bilateral Meniere disease (BMD)**. **(A)** Pie chart showing five groups or clinical variants in BMD. **(B)** Bar chart ranking the importance of predictors to define the groups. **(C)** Classification of BMD in five clinical variants according to its observed frequency and lead predictor: type 1, metachronic sensorineural hearing loss (SNHL); type 2, synchronic SNHL; type 3, familial Meniere disease (FMD); type 4, migraine; type 5, autoimmune disease.

We performed cluster analysis to identify groups of patients with common clinical features in BMD. Figure 3 shows the size of the clusters, the relevance of predictors, and the contribution of each predictor to define the cluster. The best predictors for clustering were autoimmune history, FMD, migraine, and the onset of hearing loss (synchronic/metachronic). Ninety-five patients remained unclassified because of incomplete clinical data.

**Figure 4:**
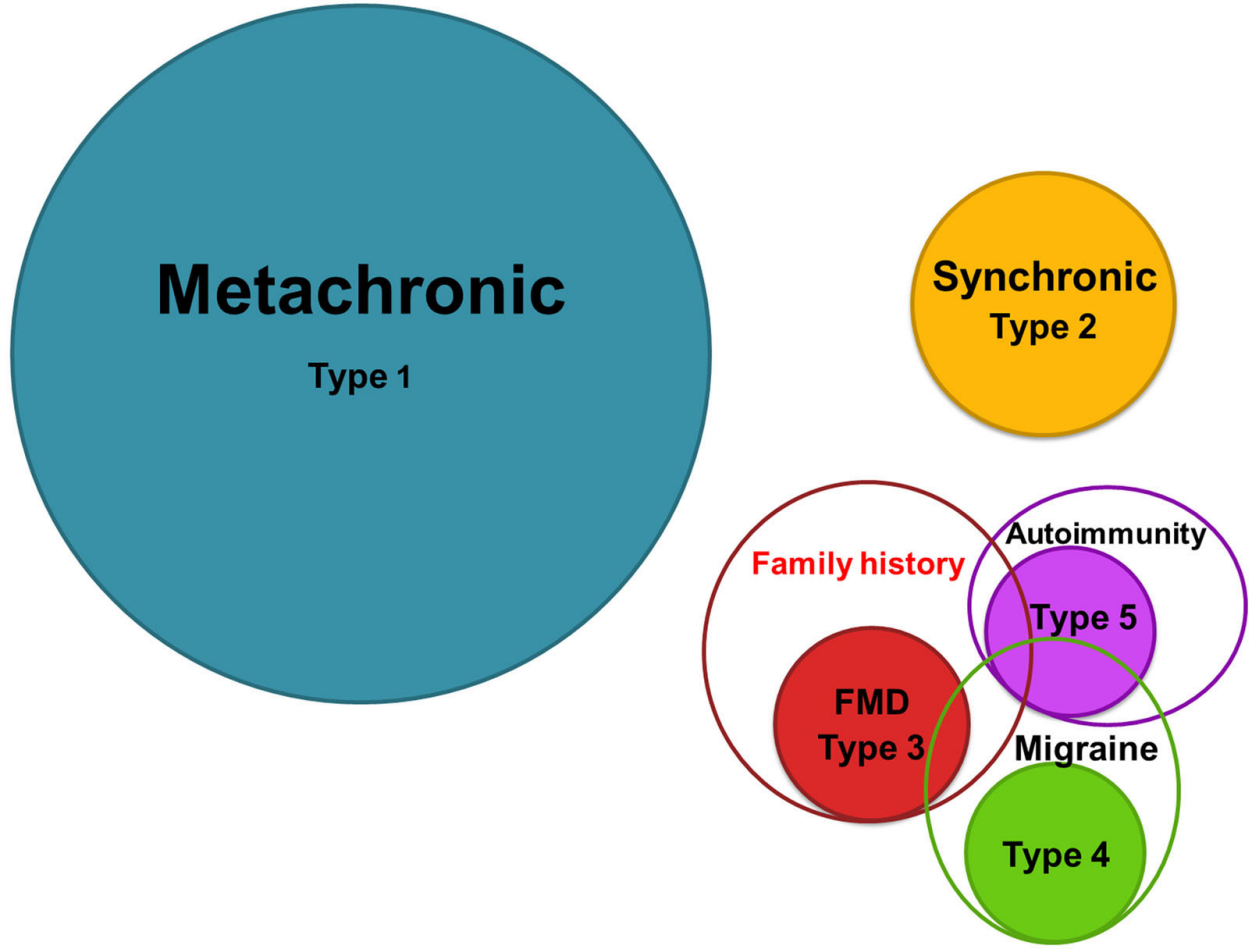
**Schematic diagram of the five subgroups in BMD**. Circle areas are proportional to the frequency observed in each group.

We have defined five clusters for BMD and ranked them according to its relative frequency (Figure 4). Cluster 1 is the most common, including 46.5% of patients, and it is defined by metachronic hearing loss without migraine, sporadic BMD, and no autoimmune history. Cluster 2 (17.5%) includes patients with synchronic hearing loss, sporadic BMD, no migraine, and no autoimmune history. Cluster 3 (12.9%) includes patients with FMD without migraine in 82% of patients. Cluster 4 (11.9%) consists of patients with migraine and sporadic BMD. Cluster 5 (11.2%) groups all patients with autoimmune comorbidities, being 71% sporadic and 29% FMD.

Table 6 shows the five groups found and the major clinical differences among the groups. Comparing the age of onset by groups, we observe that groups 3–5 have earlier onsets than groups 1 and 2 (*p* = 0.0003). The type of hearing loss, FMD, migraine, and autoimmune comorbidities strongly differ among groups, and these variables are the basis to assign a given patient to each cluster.

**Table 6:** **Clinical variants in bilateral Meniere disease (BMD) defined by two-step cluster analysis**.

Variables	BMD type 1 (*n* = 141)	BMD type 2 (*n* = 53)	BMD type 3 |(*n* = 39)	BMD type 4 (*n* = 36)	BMD type 5 (*n* = 34)	*p*-value

Group predictor	Metachronic SNHL	Synchronic SNHL	FMD	Migraine	AD	
Age, mean (SD)	63.3 (11.0)	62.4 (9.5)	54.7 (13.2)	54.1 (11.5)	59.7 (11.1)	**0.00001**
Gender (% women)	73 (51.8)	30 (56.6)	26 (66.7)	25 (69.4)	24 (70.6)	0.11
Age of onset (SD)	46.4 (13.1)	47.9 (12.0)	40 (14.5)	**37 (12.5)**	39.8 (11.3)	**0.0003**
Age of onset ≤40, *n* (%)	46 (32.6)	15 (28.3)	19 (48.7)	21 (58.3)	16 (47.1)	**0.011**
Synchronic, *n* (%)	0 (0.0)	53 (100.0)	7 (17.9)	8 (22.2)	13 (38.2)	**3.39 × 10**^−^42^^
Metachronic, *n* (%)	141 (100.0)	0 (0.0)	32 (82.1)	28 (77.8)	21 (61.8)	
Family history, *n* (%)	18 (12.8)	7 (13.2)	39 (100.0)	7 (19.4)	19 (55.9)	**1.81 × 10^−27^**
FMD, *n* (%)	0 (0.0)	0 (0.0)	39 (100.0)	0 (0.0)	10 (29.4)	**4.10 × 10^−53^**
Headache, *n* (%)	22 (15.6)	20 (37.7)	14 (35.9)	36 (100.0)	23 (67.6)	**4.88 × 10^−20^**
Migraine, *n* (%)	0 (0.0)	0 (0.0)	7 (17.9)	36 (100.0)	13 (38.2)	**1.21 × 10^−44^**
Rheumatoid history, *n* (%)	0 (0.0)	0 (0.0)	0 (0.0)	0 (0.0)	34 (100.0)	**2.44 × 10^−64^**
Cardiovascular risk factors						
High blood pressure, *n* (%)	46 (34.3)	23 (50.0)	10 (27.0)	11 (34.4)	15 (46.9)	0.15
Dyslipidemia, *n* (%)	58 (45.3)	26 (53.1)	16 (42.1)	13 (43.3)	15 (45.5)	0.86
Type 2 diabetes, *n* (%)	23 (17.8)	9 (18.4)	7 (18.4)	1 (3.2)	12 (36.4)	**0.019**
Smoking, *n* (%)	31 (22.6)	10 (18.9)	12 (30.8)	6 (17.1)	9 (29.0)	0.53

*Significant p values in bold*.

## Discussion

The diagnostic criteria for MD formulated by the Classification Committee of the Bárány Society state that bilateral involvement is determined by hearing loss defined in the audiogram (1). So, if the absolute thresholds for bone-conducted sound are ≥35 dB HL at each of two contiguous frequencies below 2000 Hz in both ears, and the patient has experienced ≥2 episodes of spontaneous vertigo each lasting 20 min to 12 h associated with fluctuating aural symptoms, the diagnosis of definite BMD is established. The notes added to the definition also describe a second clinical variant when the patient develops simultaneous bilateral SNHL (symmetric or asymmetric) (1, 52), but no further clinical information was included in the definition.

Our study demonstrates that BMD is a heterogeneous disorder, and two-step cluster analysis is a very useful tool to define groups of patients with BMD according to four clinical predictors: FMD, autoimmune history, migraine, and the type of onset for hearing loss. We selected this method since it allows the inclusion of quantitative and categorical variables to define clusters (53).

We present a new classification for BMD in five groups of patients with potential etiological implications, which probably will improve the diagnostic workflow and the management of patients with BMD. Previous studies in patients with BMD were focused in the diagnosis by electrocochleography or MRI (54–56), but they did not consider the comorbidities commonly observed, such as migraine or AD in some cases. The phenotype of a patient with an episodic vestibular syndrome should not be limited to the description of the inner ear symptoms, skipping crucial information such as the familiar history of MD or migraine. Furthermore, the comorbidities of migraine or AD may explain the perception of MD as a continuum, which overlaps with migraine (57) or autoimmune inner ear disease (1, 58, 59).

The most remarkable finding in our study is that the five groups of patients identified do not overlap themselves, and each of them has a set of features able to define the group.

Bilateral MD type 1 is the most common clinical variant, and it includes patients with MD in one ear (unilateral MD), and they develop the hearing loss in the contralateral ear (conversion from unilateral to BMD). The mean age of onset was 46 years old, comparable to BMD type 2, but it is significantly higher than it was observed in the rest of the groups (types 3, 4, or 5). BMD type 1 has no familial or autoimmune history, and patients do not have migraine, so further studies are required to investigate other concurrent comorbidities to determine contributing factors.

Bilateral MD type 2 is the second most frequently observed clinical variant, and fluctuating bilateral SNHL loss may resemble AIED, since simultaneous SNHL with vestibular symptoms can occur in 50% patients with AIED (58). However, these patients do not have any autoimmune comorbid conditions, migraine, or familial history of MD. Interestingly, BMD type 2 patients show a vascular risk profile, since 50% of them show high blood pressure, and 53% have dyslipidemia. When we compared these frequencies with BMD type 1, which do not differ in age or sex profile to BMD type 2, they were not significantly different (*p* = 0.078), but further studies should assess the role of vascular risk factors in labyrinthine microcirculation in MD.

Comparing the hearing stage for the worst ear, it seems to be worse in BMD type 1 (metachronic SNHL) than in type 2 (synchronic SNLH). Since both groups do not differ for the age of onset, duration of disease, or gender distribution, we cannot determine the reason for the severe SNHL in the first ear in BMD type 1.

Bilateral MD type 3 includes all patients with familiar history of MD, and we could subtype them in two subgroups (3a with migraine, 82%, and 3b BMD without migraine 18%). These findings confirm the early description of families with MD co-segregating migraine and MD (60) and the more recent description of FMD without migraine (2, 61, 62). According to this subtyping for FMD, there will be two types of families with MD, with and without migraine, and they reflect the genetic heterogeneity in FMD. The families include patients with uni and BMD, so epigenetic factors may influence uni or bilateral involvement. Most of the described families have an autosomal dominant pattern of inheritance, and the participation of several genes indicate a genetic heterogeneity in FMD (2, 50). Although variable expressivity and incomplete penetrance was observed, we did not find cases with episodic ataxia in the families.

Bilateral MD type 4 is associated with migraine in all cases, but they do not have familial history of MD. This group may overlap with VM, and it may share common pathophysiological mechanisms (63). Patients with MD may show migraine symptoms even during the attacks of vertigo (57), and this finding could make difficult the differential diagnosis of VM and MD. Magnetic resonance imaging may be useful in the diagnostic evaluation of patients with the spectrum of VM/MD (MD with concurrent migraine or in cases VM and auditory symptoms) (64).

Bilateral MD type 5 could be considered as autoimmune MD, since all patients have another concurrent AD. However, this group is heterogeneous and includes patients with sporadic (71%), FMD (29%), migraine (38%), and both synchronic (38%) and metachronic SNHL (62%). Patients with BMD type 5 and migraine may have either synchronic or metachronic SNHL.

Our study has several limitations. Despite our efforts to improve phenotyping in patients with BMD, we could not classify 95 patients with BMD in any cluster, and they were excluded of the model. In fact, the largest group (BMD type 1) remains poorly characterized, since it is not associated with any particular clinical feature or etiological factor. The role of allergy in MD deserves more research efforts, since a high prevalence of sensitization to inhalant or food allergies have been reported in MD (65–67).

However, the recognizing of different subgroups of patients and the definition of clinical variants in BMD is not only the first step to improve the selection of patients for genetic and immunological studies but also for randomized clinical trials (RCT). Most of the RCT performed in MD, were not able to demonstrate any effects of diuretics (68) or betahistine (69) and had limited effectiveness for intratympanic gentamicin (70) or steroids (71), and these results could be explained by a biased selection of patients with different etiologies. Further phenotyping of these clinical variants are needed for a better understanding of the clinical heterogeneity observed in BMD.

## Author Contributions

LF and JL-E conceived and designed the study. AS-V, SS-P, AB-C, VP-G, HP-G, JF, EM-S, MT, GT, AG-A, RG-A, JE-S, PM, PP, JB, and JL-E collected clinical information. LF and JL-E analyzed the data and drafted the manuscript. LF, AS-V, SS-P, AB-C, VP-G, HP-G, JF, EM-S, MT, GT, AG-A, RG-A, JE-S, PM, PP, JB, and JL-E revised and approved the final version of the manuscript.

## Conflict of Interest Statement

The authors declare that the research was conducted in the absence of any commercial or financial relationships that could be construed as a potential conflict of interest.

## References

[1] Lopez-Escamez JA Carey J Chung WH Goebel JA Magnusson M Mandala M Diagnostic criteria for Meniere’s disease. J Vestib Res (2015) 25(1):1–7. 10.3233/VES-150549 25882471

[2] Requena T Espinosa-Sanchez JM Cabrera S Trinidad G Soto-Varela A Santos-Perez S Familial clustering and genetic heterogeneity in Meniere’s disease. Clin Genet (2014) 85(3):245–52. 10.1111/cge.12150 23521103

[3] Stahle J Friberg U Svedberg A Long-term progression of Meniere’s disease. Am J Otol (1989) 10(3):170–3. 2750865

[4] Belinchon A Perez-Garrigues H Tenias JM Evolution of symptoms in Meniere’s disease. Audiol Neurootol (2012) 17(2):126–32. 10.1159/000331945 21985844

[5] Perez-Garrigues H Lopez-Escamez JA Perez P Sanz R Orts M Marco J Time course of episodes of definitive vertigo in Meniere’s disease. Arch Otolaryngol Head Neck Surg (2008) 134(11):1149–54. 10.1001/archotol.134.11.1149 19015442

[6] Gazquez I Soto-Varela A Aran I Santos S Batuecas A Trinidad G High prevalence of systemic autoimmune diseases in patients with Meniere’s disease. PLoS One (2011) 6(10):e26759 10.1371/journal.pone.0026759 22053211 PMC3203881

[7] Huppert D Strupp M Brandt T Long-term course of Meniere’s disease revisited. Acta Otolaryngol (2010) 130(6):644–51. 10.3109/00016480903382808 20001444

[8] House JW Doherty JK Fisher LM Derebery MJ Berliner KI. Meniere’s disease: prevalence of contralateral ear involvement. Otol Neurotol (2006) 27(3):355–61. 10.1097/00129492-200604000-00011 16639274

[9] Lopez-Escamez JA Viciana D Garrido-Fernandez P. Impact of bilaterality and headache on health-related quality of life in Meniere’s disease. Ann Otol Rhinol Laryngol (2009) 118(6):409–16. 19663372 10.1177/000348940911800603

[10] Kitahara M Matsubara H Takeda T Yazawa Y. Bilateral Meniere’s disease. Adv Otorhinolaryngol (1979) 25:117–21. 10.1159/000402927 484343

[11] Paparella MM Griebie MS. Bilaterality of Meniere’s disease. Acta Otolaryngol (1984) 97(3–4):233–7. 10.3109/00016488409130984 6720298

[12] Rosenberg S Silverstein H Flanzer J Wanamaker H Bilateral Meniere’s disease in surgical versus nonsurgical patients. Am J Otol (1991) 12(5):336–40. 1789301

[13] Palaskas CW Dobie RA Snyder JM Progression of hearing loss in bilateral Meniere’s disease. Laryngoscope (1988) 98(3):287–90. 10.1288/00005537-198803000-00009 3343878

[14] Tokumasu K Fujino A Yoshio S Hoshino I Prognosis of Meniere’s disease by conservative treatment: retrospective study on the time course of the disease. Acta Otolaryngol Suppl (1995) 519:216–8. 10.3109/00016489509121908 7610872

[15] Tokumasu K Fujino A Naganuma H Hoshino I Arai M Initial symptoms and retrospective evaluation of prognosis in Meniere’s disease. Acta Otolaryngol Suppl (1996) 524:43–9. 10.3109/00016489609124348 8790762

[16] Chaves AG Boari L Lei Munhoz MS The outcome of patients with Menieres disease. Braz J Otorhinolaryngol (2007) 73(3):346–50. 10.1016/S1808-8694(15)30078-1 17684655 PMC9445754

[17] Balkany TJ Sires B Arenberg IK Bilateral aspects of Meniere’s disease: an underestimated clinical entity. Otolaryngol Clin North Am (1980) 13(4):603–9. 7454321

[18] Green JD Jr Blum DJ Harner SG Longitudinal followup of patients with Meniere’s disease. Otolaryngol Head Neck Surg (1991) 104(6):783–8. 10.1177/019459989110400603 1908968

[19] Stahle J Friberg U Svedberg A Long-term progression of Meniere’s disease. Acta Otolaryngol Suppl (1991) 485:78–83. 10.3109/00016489109128047 1843175

[20] Havia M Kentala E Progression of symptoms of dizziness in Meniere’s disease. Arch Otolaryngol Head Neck Surg (2004) 130(4):431–5. 10.1001/archotol.130.4.431 15096425

[21] Friberg U Stahle J Svedberg A. The natural course of Meniere’s disease. Acta Otolaryngol Suppl (1984) 406:72–7. 6591717 10.3109/00016488309123007

[22] Morrison AW. Predictive tests for Meniere’s disease. Am J Otol (1986) 7(1):5–10. 3946582

[23] Tyrrell JS Whinney DJ Ukoumunne OC Fleming LE Osborne NJ Prevalence, associated factors, and comorbid conditions for Meniere’s disease. Ear Hear (2014) 35(4):e162–9. 10.1097/AUD.0000000000000041 24732693

[24] Kim SH Kim JY Lee HJ Gi M Kim BG Choi JY. Autoimmunity as a candidate for the etiopathogenesis of Meniere’s disease: detection of autoimmune reactions and diagnostic biomarker candidate. PLoS One (2014) 9(10):e111039. 10.1371/journal.pone.0111039 25330336 PMC4201580

[25] Chiarella G Saccomanno M Scumaci D Gaspari M Faniello MC Quaresima B Proteomics in Meniere disease. J Cell Physiol (2012) 227(1):308–12. 10.1002/jcp.22737 21437900

[26] Chiarella G Di Domenico M Petrolo C Saccomanno M Rothenberger R Giordano A A proteomics-driven assay defines specific plasma protein signatures in different stages of Meniere’s disease. J Cell Biochem (2014) 115(6):1097–100. 10.1002/jcb.24747 24356812

[27] Lopez-Escamez JA Saenz-Lopez P Gazquez I Moreno A Gonzalez-Oller C Soto-Varela A Polymorphisms of CD16A and CD32 Fcgamma receptors and circulating immune complexes in Meniere’s disease: a case-control study. BMC Med Genet (2011) 12:2 10.1186/1471-2350-12-2 21208440 PMC3022798

[28] Brand O Gough S Heward J. HLA, CTLA-4 and PTPN22: the shared genetic master-key to autoimmunity? Expert Rev Mol Med (2005) 7(23):1–15. 10.1017/S1462399405009981 16229750

[29] McCabe BF. Autoimmune sensorineural hearing loss. Ann Otol Rhinol Laryngol (1979) 88(5 Pt 1):585–9. 10.1177/000348947908800501 496191

[30] Amor-Dorado JC Paco L Martin J Lopez-Nevot MA Gonzalez-Gay MA. Human leukocyte antigen-DQB1 and -DRB1 associations in patients with idiopathic sudden sensorineural hearing loss from a defined population of Northwest Spain. Acta Otolaryngol (2005) 125(12):1277–82. 10.1080/00016480510012228 16303674

[31] Harris JP Weisman MH Derebery JM Espeland MA Gantz BJ Gulya AJ Treatment of corticosteroid-responsive autoimmune inner ear disease with methotrexate: a randomized controlled trial. JAMA (2003) 290(14):1875–83. 10.1001/jama.290.14.1875 14532316

[32] Hughes GB Kinney SE Barna BP Calabrese LH Autoimmune reactivity in Meniere’s disease: a preliminary report. Laryngoscope (1983) 93(4):410–7. 6834965 10.1002/lary.1983.93.4.410

[33] Yoo TJ Shea J Jr Ge X Kwon SS Yazawa Y Sener O Presence of autoantibodies in the sera of Meniere’s disease. Ann Otol Rhinol Laryngol (2001) 110(5 Pt 1):425–9. 10.1177/000348940111000506 11372925

[34] Fattori B Nacci A Dardano A Dallan I Grosso M Traino C Possible association between thyroid autoimmunity and Meniere’s disease. Clin Exp Immunol (2008) 152(1):28–32. 10.1111/j.1365-2249.2008.03595.x 18241228 PMC2384067

[35] Furuta T Teranishi M Uchida Y Nishio N Kato K Otake H Association of interleukin-1 gene polymorphisms with sudden sensorineural hearing loss and Meniere’s disease. Int J Immunogenet (2011) 38(3):249–54. 10.1111/j.1744-313X.2011.01004.x 21385326

[36] Gazquez I Moreno A Requena T Ohmen J Santos-Perez S Aran I Functional variants of MIF, INFG and TFNA genes are not associated with disease susceptibility or hearing loss progression in patients with Meniere’s disease. Eur Arch Otorhinolaryngol (2013) 270(4):1521–9. 10.1007/s00405-012-2268-0 23179933

[37] Lopez-Escamez JA Saenz-Lopez P Acosta L Moreno A Gazquez I Perez-Garrigues H Association of a functional polymorphism of PTPN22 encoding a lymphoid protein phosphatase in bilateral Meniere’s disease. Laryngoscope (2010) 120(1):103–7. 10.1002/lary.20650 19780033

[38] Cabrera S Sanchez E Requena T Martinez-Bueno M Benitez J Perez N Intronic variants in the NFKB1 gene may influence hearing forecast in patients with unilateral sensorineural hearing loss in Meniere’s disease. PLoS One (2014) 9(11):e112171. 10.1371/journal.pone.0112171 25397881 PMC4232390

[39] Gazquez I Moreno A Aran I Soto-Varela A Santos S Perez-Garrigues H MICA-STR A.4 is associated with slower hearing loss progression in patients with Meniere’s disease. Otol Neurotol (2012) 33(2):223–9. 10.1097/MAO.0b013e31824296c8 22222578

[40] Requena T Gazquez I Moreno A Batuecas A Aran I Soto-Varela A Allelic variants in TLR10 gene may influence bilateral affectation and clinical course of Meniere’s disease. Immunogenetics (2013) 65(5):345–55. 10.1007/s00251-013-0683-z 23370977

[41] Radtke A Lempert T Gresty MA Brookes GB Bronstein AM Neuhauser H Migraine and Meniere’s disease: is there a link? Neurology (2002) 59(11):1700–4. 10.1212/01.WNL.0000036903.22461.39 12473755

[42] Lempert T Olesen J Furman J Waterston J Seemungal B Carey J Vestibular migraine: diagnostic criteria. J Vestib Res (2012) 22(4):167–72. 10.3233/VES-2012-0453 23142830

[43] Neff BA Staab JP Eggers SD Carlson ML Schmitt WR Van Abel KM Auditory and vestibular symptoms and chronic subjective dizziness in patients with Meniere’s disease, vestibular migraine, and Meniere’s disease with concomitant vestibular migraine. Otol Neurotol (2012) 33(7):1235–44. 10.1097/MAO.0b013e31825d644a 22801040

[44] Brown MR The factor of heredity in labyrinthine deafness and paroxysmal vertigo; Meniere’s syndrome. Ann Otol Rhinol Laryngol (1949) 58(3):665–70. 15397195 10.1177/000348944905800303

[45] Morrison AW Bailey ME Morrison GA Familial Meniere’s disease: clinical and genetic aspects. J Laryngol Otol (2009) 123(1):29–37. 10.1017/S0022215108002788 18616841

[46] Requena T Espinosa-Sanchez JM Lopez-Escamez JA. Genetics of dizziness: cerebellar and vestibular disorders. Curr Opin Neurol (2014) 27(1):98–104. 10.1097/WCO.0000000000000053 24275721

[47] Frejo L Giegling I Teggi R Lopez-Escamez JA Rujescu D. Genetics of vestibular disorders: pathophysiological insights. J Neurol (2016) 263(Suppl 1):45–53. 10.1007/s00415-015-7988-9 27083884 PMC4833787

[48] Lee JM Kim MJ Jung J Kim HJ Seo YJ Kim SH. Genetic aspects and clinical characteristics of familial Meniere’s disease in a South Korean population. Laryngoscope (2015) 125(9):2175–80. 10.1002/lary.25207 25946228

[49] Ohmen JD White CH Li X Wang J Fisher LM Zhang H Genetic evidence for an ethnic diversity in the susceptibility to Meniere’s disease. Otol Neurotol (2013) 34(7):1336–41. 10.1097/MAO.0b013e3182868818 23598705

[50] Requena T Cabrera S Martin-Sierra C Price SD Lysakowski A Lopez-Escamez JA. Identification of two novel mutations in FAM136A and DTNA genes in autosomal-dominant familial Meniere’s disease. Hum Mol Genet (2015) 24(4):1119–26. 10.1093/hmg/ddu524 25305078 PMC4834881

[51] Committee on Hearing and Equilibrium guidelines for the diagnosis and evaluation of therapy in Meniere’s disease. American Academy of Otolaryngology-Head and Neck Foundation, Inc. Otolaryngol Head Neck Surg (1995) 113(3):181–5. 10.1016/S0194-5998(95)70102-8 7675476

[52] Belinchon A Perez-Garrigues H Tenias JM Lopez A Hearing assessment in Meniere’s disease. Laryngoscope (2011) 121(3):622–6. 10.1002/lary.21335 21305548

[53] Tyler R Coelho C Tao P Ji H Noble W Gehringer A Identifying tinnitus subgroups with cluster analysis. Am J Audiol (2008) 17(2):S176–84. 10.1044/1059-0889(2008/07-0044) 19056922 PMC2668860

[54] Nabi S Parnes LS Bilateral Meniere’s disease. Curr Opin Otolaryngol Head Neck Surg (2009) 17(5):356–62. 10.1097/MOO.0b013e3283304cb3 19617826

[55] Morita N Kariya S Farajzadeh Deroee A Cureoglu S Nomiya S Nomiya R Membranous labyrinth volumes in normal ears and Meniere disease: a three-dimensional reconstruction study. Laryngoscope (2009) 119(11):2216–20. 10.1002/lary.20723 19806642 PMC2927481

[56] Nonoyama H Tanigawa T Tamaki T Tanaka H Yamamuro O Ueda H. Evidence for bilateral endolymphatic hydrops in ipsilateral delayed endolymphatic hydrops: preliminary results from examination of five cases. Acta Otolaryngol (2014) 134(3):221–6. 10.3109/00016489.2013.850741 24279647

[57] Lopez-Escamez JA Dlugaiczyk J Jacobs J Lempert T Teggi R von Brevern M Accompanying symptoms overlap during attacks in Meniere’s disease and vestibular migraine. Front Neurol (2014) 5:265 10.3389/fneur.2014.00265 25566172 PMC4265699

[58] Pathak S Hatam LJ Bonagura V Vambutas A. Innate immune recognition of molds and homology to the inner ear protein, cochlin, in patients with autoimmune inner ear disease. J Clin Immunol (2013) 33(7):1204–15. 10.1007/s10875-013-9926-x 23912888 PMC3809107

[59] Eisen MD Niparko JK Chapter 31 – autoimmune inner ear disease. In: Eggers SDZ Zee DS, editors. Handbook of Clinical Neurophysiology (Vol. 9), Elsevier (2010). p. 428–32.

[60] Oliveira CA Ferrari I Messias CI Occurrence of familial Meniere’s syndrome and migraine in Brasilia. Ann Otol Rhinol Laryngol (2002) 111(3 Pt 1):229–36. 10.1177/000348940211100307 11913683

[61] Arweiler-Harbeck D Horsthemke B Jahnke K Hennies HC Genetic aspects of familial Meniere’s disease. Otol Neurotol (2011) 32(4):695–700. 10.1097/MAO.0b013e318216074a 21436747

[62] Hietikko E Kotimaki J Kentala E Klockars T Sorri M Mannikko M. Finnish familial Meniere disease is not linked to chromosome 12p12.3, and anticipation and cosegregation with migraine are not common findings. Genet Med (2011) 13(5):415–20. 10.1097/GIM.0b013e3182091a41 21346584

[63] Espinosa-Sanchez JM Lopez-Escamez JA New Insights into pathophysiology of vestibular migraine. Front Neurol (2015) 6:12 10.3389/fneur.2015.00012 25705201 PMC4319397

[64] Gurkov R Kantner C Strupp M Flatz W Krause E Ertl-Wagner B. Endolymphatic hydrops in patients with vestibular migraine and auditory symptoms. Eur Arch Otorhinolaryngol (2014) 271(10):2661–7. 10.1007/s00405-013-2751-2 24121780

[65] Di Berardino F Cesarani A. Gluten sensitivity in Meniere’s disease. Laryngoscope (2012) 122(3):700–2. 10.1002/lary.22492 22253033

[66] Derebery MJ Allergic and immunologic features of Meniere’s disease. Otolaryngol Clin North Am (2011) 44(3):655–66, ix. 10.1016/j.otc.2011.03.004 21621052

[67] Powers WH Allergic factors in Meniere’s disease. Trans Am Acad Ophthalmol Otolaryngol (1973) 77(1):ORL22–9. 4730087

[68] Thirlwall AS Kundu S Diuretics for Meniere’s disease or syndrome. Cochrane Database Syst Rev (2006) (3):CD003599 10.1002/14651858.CD003599.pub2 16856015 PMC9007146

[69] Adrion C Fischer CS Wagner J Gurkov R Mansmann U Strupp M Efficacy and safety of betahistine treatment in patients with Meniere’s disease: primary results of a long term, multicentre, double blind, randomised, placebo controlled, dose defining trial (BEMED trial). BMJ (2016) 352:h6816. 10.1136/bmj.h6816 26797774 PMC4721211

[70] Pullens B van Benthem PP Intratympanic gentamicin for Meniere’s disease or syndrome. Cochrane Database Syst Rev (2011) (3):CD008234 10.1002/14651858.CD008234.pub2 21412917 PMC13378876

[71] Phillips JS Westerberg B Intratympanic steroids for Meniere’s disease or syndrome. Cochrane Database Syst Rev (2011) (7):CD008514 10.1002/14651858.CD008514.pub2 21735432 PMC13378880

